# Incidence of inherited metabolic disorders in southern Israel: a comparison between consanguinity and non-consanguinity communities

**DOI:** 10.1186/s13023-020-01578-3

**Published:** 2020-11-25

**Authors:** G. Hazan, E. Hershkovitz, O. Staretz-Chacham

**Affiliations:** 1 Division of Pediatrics, Soroka University Medical Center, Ben Gurion University, Beer Sheva, Israel; 2 Metabolic Clinic, Soroka University Medical Center, Ben Gurion University, 151 Rager Ave., Beer Sheva, Israel; 3 Neonatlogy Unit, Soroka University Medical Center, Ben Gurion University, Beer Sheva, Israel

**Keywords:** Inherited metabolic diseases, Consanguinity, Jewish, Bedouin-muslim, Prevalence

## Abstract

**Background:**

Inherited metabolic disorders (IMDs) are group of rare monogenic diseases, usually derived from reduced or absent activity in a single metabolic pathway. Most of the IMDs are inherited in an autosomal recessive manner. The incidence of IMDs varies from country to country and within different ethnic groups, but data is still scarce. Consanguinity rate among populations is highly contributor factor for IMDs incidence. There are no reports comparing the incidence of IMD in consanguineous and non-consanguineous populations from the same geographic region with the same diagnostic capabilities. Our study objective is to compare the incidence of IMDs between between the relatively low consanguineous Jewish population and the consanguineous Bedouin population, both living in the southern of Israel.

**Results:**

During 1990–2017 there were 393,772 live births in the Negev district, of Southern of Israel. Among them 187,049 were of Jewish origin while 206,723 were of Bedouin-Muslim origin. A total of 223 children were diagnosed in this study period with IMDs. Among those 223 children with IMD, 33 were of Jewish origin while the other 190 children were of Bedouin-Muslim origin. The overall incidence for IMDs of the overall Negev population was 56.6/100,000 live birth. The incidence for IMD's among the Bedouin population was significantly higher than among Jewish population.

**Conclusions:**

IMDs are extremely more common in the consanguineous Bedouin compared with the relatively non-consanguineous Jewish population of Southern Israel. Health policy makers should consider these data and prepare educational and genetic counselling problems accordingly.

## Background

Inherited metabolic disorders (IMD's) comprise a heterogeneous group of over 500 rare monogenic diseases, mostly derived from reduced or absent activity in a single metabolic pathway [1, 2]. Approximately 80% of the IMD are inherited in an autosomal recessive manner [3]. The clinical outcomes of IMD are often severe, although, disease progression and deterioration may be related to delayed timing of the appropriate diagnosis [4, 5]. There might be an overlap of clinical presentation between different diseases, hence diagnoses of IMD can be challenging.

Reported incidence of IMDs in the literature varies from country to country and even within different regions in the same country. Although the incidence of each disorder is rare, their cumulative incidence is substantial and the quoted one is 20–40 per 10,0000 live birth [5, 6]. Despite numerous reports of cumulative incidence of IMD in different countries such as 15.7/100,000 live birth in Australia; 27/100,000 in Italy; 1:2500 in Canada; whilst in West Midlands region in United Kingdom it reaches up to 1:784 [7–10], there is still lack of data, globally and regionally, about the true incidence of IMD, resulting in difficulty in planning and providing appropriate clinical services for the patients. Nowadays with the development of new technologies for diagnosis, screening and treatment the importance of these issues is more prominent [11–18].

As described previously, the inheritance pattern of most IMDs is by Autosomal Recessive manner. Hence, in countries and regions with high consanguinity rates, the incidence of IMDs and other rare disorders is increased [19]. Coefficient of Inbreeding is the probability that two genes at any locus in an individual are identical by descent from the common ancestor(s) of the two parents [20]. The coefficient of inbreeding range from 0.00004–0.00008 in Canada, while in Saudi Arabia, where the consanguinity is as high, as 40% among first cousins and up to 60% interfamilial marriages, the coefficient of inbreeding is 0.024 [21–23] resulting in a reported incidence of IMDs in Saudi Arabia of 150/100,000 live birth [24]. This incidence can be influenced by a high index of suspicion for IMD in Saudi Arabia. So far, there are no reports comparing the incidence of IMD in consanguineous and non-consanguineous populations residing in the same geographic region and exposed to the same diagnostic capabilities.

Southern Israel is a heterogeneously populated area, inhabited by two major populations—Jewish and Bedouin (Muslim). Consanguinity is highly sociocultural-related. Amongst Arab population and especially the Bedouin population there are high rates of consanguinity, similar to tribes in Saudi Arabia [25] and also the tendency to have more children per family. Approximately 45% of marriages in the Bedouin population in Israel are consanguineous, most of them are first cousin origin [26]. The consanguinity in the Jewish population on the other hand is relatively low.

Our study aimed to calculate the incidences of various IMDs in southern Israel with comparison of the incidence between the relatively low consanguineous Jewish population and the consanguineous Bedouin population.

## Results

Over 27 years of study period there were 393,772 live births in the Negev district of Southern Israel. Among them 187,049(48%) were of Jewish origin while 206,723(52%) were of Bedouin-Muslim origin. A total of 223 children were diagnosed in this study period with IMDs. All of those children were diagnosed in Soroka University Medical Center by clinical suspicion and/or laboratory testing in hospital or by the Israeli newborn screening program. Among those 223 children with IMD, 33(15%) were of Jewish origin while the other 190(85%) children were of Bedouin-Muslim origin. The overall incidence for IMDs of the Negev population was 56.6/100,000 live birth. The incidence for IMD's among the Bedouin population was significantly higher than among Jewish population (101.6/100,000 Vs 16/100,000, respectively, *P* value < 0.001) (Table 1**)**.

**Table 1:** Incidence of inherited metabolic diseases categories among Jews versus Bedouin-Muslim population, in southern of Israel, between the years 1990 and 2017

Disease category	Overall incidence/100,000 live births	Incidence among Bedouin-Muslim/100,000 live births	Incidence among Jews/100,000 live births	*P* value
Overall	56.6	101.6	16	< 0.001
Aminoacidopathy	8.6	18.2	0	< 0.001
Peroxisomal diseases	5.3	9.1	1.9	< 0.001
Sphingolipidosis	4.8	9.1	1	< 0.001
Organic aciduria	2	4.3	0	0.003
Fatty acid oxidation diseases	4.8	7	2.9	0.02
Mucopolysaccharidosis	6.9	12.8	1.5	< 0.001
Glycogen storage diseases*	11.2	15	7.7	0.03
Pompe disease (type 2 Glycogen storage disease)	2.3	4.8	0	0.003
Mitochondrial diseases	10.7	21.4	1	< 0.001

Birth incidence for all IMD's categories was significantly higher among the Bedouin-Muslim population in comparison with the Jewish population, as presented in Table 1. The incidence of Peroxisomal diseases for Bedouin-Muslims per 100,000 live births was 9.1 while for Jewish it was 1.9. Similar differences were demonstrated for Sphingolipidoses (9.1/100,000 for Muslims-Bedouin vs. 1/100,000 for Jewish), Fatty Acid Oxidation Defects (7/100,000 vs. 2.9/100,000), Mucopolysaccharidosis (12.8/100,000 vs. 1.5/100,000) and Glycogen Storage Diseases (15/100,000 vs. 7.7/100,000). For Aminoacidurias, Organic acidurias and Pompe disease there were no affected Jewish children while the incidence of Bedouin-Muslims with disease was 18.2/100,000, 4.3/100,000 and 4.8/100,000, respectively.

Table 2 presents the births incidences for each IMD. For most of the diseases’ subtypes the incidence among the Bedouin-Muslim population was higher compared to the Jewish population. The only disease with statistically significant higher incidence among the Jewish population was Glycogen Storage disease type 3A with an incidence of 3.7/100,000 live births among Jewish patients vs. no affected patients among the Bedouin-Muslims population and the second disease with much higher incidence among Jewish patients, though not statistically significant, is Glycogen Storage disease type 1A with an incidence of 2.9/100,000 live births among Jewish patients Vs. no affected patients among the Bedouin-Muslims population. Most of the other mentioned IMDs were statistically significant higher among the Bedouin-Muslims population.

**Table 2:** Incidence of each inherited metabolic disease among Jews versus Bedouin-Muslim population, in southern of Israel, between the years 1990 and 2017

Disease category	Overall incidence/100,000 live births	Incidence among Bedouin-Muslim/100,000 live births	Incidence among Jews/100,000 live births	P value
*Aminoacidopathy*				
Maple syrup urine disease (MSUD)	4.8	10.2	0	< 0.001
Non-ketotic hyperglycinemia	3.8	8	0	< 0.001
*Peroxisomal diseases*				
X-linked Adrenoleukodystrophy	0.8	0	1.6	0.1
Zellweger disease	4.6	8.2	0.5	< 0.001
*Sphingolipidosis*				
Niemann Pick C type 1	4.8	9.1	1	< 0.001
*Organic aciduria*				
Glutaric aciduria type 1	2	4.3	0	0.003
*Fatty acid oxidation diseases*				
Multiple acyl-CoA dehydrogenase deficiency	0.5	1	0	0.1
Medium chain acyl-CoA dehydrogenase deficiency	1	0.5	1.5	0.4
Very long chain acyl-CoA dehydrogenase deficiency	2.3	4.3	0.5	0.01
Carnitine palmitoyl-transferase 1A	0.3	0.5	0	0.3
Carnitine palmitoyl- transferase 2	0.5	0	1	0.2
Long chain 3-hydroxyl-CoA dehydrogenase deficiency	0.5	0.3	0	0.3
*Mucopolysaccharidosis*				
Mucopolysaccharidosis type 1	1	1.1	1	0.9
Mucopolysaccharidosis type 3	2	3.7	0.5	0.02
Mucopolysaccharidosis type 3	2.8	5.9	0	< 0.001
Unclassified mucopolysaccharidosis	0.8	1.1	0.5	0.6
*Glycogen storage diseases*				
Glycogen storage disease type 0	0.3	0	0.5	0.3
Glycogen storage disease type 1A	1.5	0	2.9	0.02
Glycogen Storage disease type 1B	4.1	8	0.5	< 0.001
Glycogen storage disease type IIIA	1.8	0	3.7	< 0.001
Glycogen storage disease type 6	2.3	4.8	0	< 0.001
Glycogen storage disease type 9	0.3	0.5	0	0.3
Glycogen storage disease type 11	0.3	0.5	0	0.3
Unclassified glycogen storage disease	0.8	1.1	0.5	0.6
Pompe disease (type 2 glycogen storage disease)	2.3	4.8	0	0.003
*Mitochondrial diseases*				
Complex 1 deficiency	2.8	5.9	0	< 0.001
Complex 3 deficiency	3.3	7	0	< 0.001
Complex 5 deficiency	1.8	3.7	0	0.005
Pyruvate dehydrogenase deficiency type 1A	0.3	0	0.5	0.3
Pyruvate dehydrogenase deficiency type E3	0.5	1.1	0	0.2
Kearns Sayre syndrome	0.3	0	0.5	0.3
Mitochondrial neuro-gastrointestinal encephalopathy disease	0.3	0.5	0	0.3
Trans-membrane protein 70 (TMEM70) deficiency	0.5	1.1	0	0.2
Mitochondrial DNA depletion	0.5	1.1	0	0.2
Unspecified mitochondrial disease	0.3	0.5	0	0.3

The incidences of other IMD that were included in this study were not statistically different between the 2 populations.

## Discussion

In this study we show that in an extremely consanguineous population the incidence of IMD is significantly higher than that of a relatively non consanguineous population. This finding is expected since 80% of IMD are inherited in an autosomal recessive pattern. Yet, these data are important. IMD can be life threatening and the therapies for such disease can be extremely expensive [27]. Health policy makers should be aware that these devastating diseases are relatively common and not rare as expected, in extremely consanguineous populations. In the Bedouin populations IMD occur in at least 1/1000 live births. Prevention of IMD is possible after genetic diagnosis and genetic counselling to at risk families and this should be encouraged. Although consanguinity is considered to be rare in developed countries, in many populations in different geographic areas, consanguinity is common, suggesting that IMD as well as other genetic diseases are extremely more common in these populations.

Although the incidence of genetic diseases reflects the prevalence of genetic mutations in the population, it is noteworthy that the Bedouin population tends to have larger families. This can also affect the total incidence of IMD shown in this study. Since our aim was to compare the "real life" incidences of IMD in these populations, we did not use statistical methods to account for the possibility of a mother having more than one affected child. As a result, we did not calculate the prevalence of genetic mutations in the populations studied.

Additionally, there is a higher rate of absent prenatal care and lower rate of pregnancy termination, even with diagnosed devastating diseases, in the Bedouin population [28, 29]. In order to prevent these cases, prenatal and premarital genetic consultation should be encouraged by all the caregivers of affected families, including raising the possibilities of Pre-implantation Genetic Diagnosis (PGD).

Since the Bedouin population has a high rate of consanguinity, there is also an increased number of founder effect, and the combination of large inbreeding families with high consanguinity rate is expected to result in a higher number of, although rare, recurring mutations in this population.

The only disease with a significantly higher prevalence in the Jewish population is glycogen storage disease type IIIA. All patients are homozygous to 4,455delT in the *AGL* gene as reported by Parvari et al. [30], that share a similar phenotype and originate from Northern Africa with a calculated prevalence 1:5,400 and a carrier prevalence 1:35 amongst North African Jews which is the result of a founder effect (Table 2). Additionally, glycogen storage disease type 1 A is more common, though not significantly, in the Jewish population of Ashkenazi origin with all patients being homozygous to the same mutation- R83C in the *G6Pase* gene [31], resulting from a founder effect. The current estimated consanguinity rate amongst the Jewish population is 2.3% with an inbreeding coefficient of 0.00070 [32]. In Southern Israel, in the Negev area there are less Ashkenazi Jews than in other parts of Israel, and there is a high rate of marriage between Jews from different communities that leads to a decreased prevalence of autosomal recessive Ashkenazi diseases.

A limitation of our study is that patients’ recollection for this study had been performed in the pre and post introduction of the national newborn screening program in 2008. The Israeli Newborn Screening program includes nine IMD [29]. Since its inception, there might be an increasing diagnostic rate for the relevant diseases in our study (MCAD, VLCAD and MSUD), however, as can be seen in our study, most of the common diseases in Southern Israel are not screened in the extended screening therefore its detection rate is not expected to change between the pre and post newborn screening era. Zlotogora et al. reported of a significant increase in the number of genes with variants causing autosomal recessive diseases among Israeli Arabs in the last several years [33]. It was presumed that it is mostly the result of the availability of better diagnostic possibilities for molecular diagnosis in genes already well known. Therefore, we should take into consideration that prior to this screening the diagnosed patients were those with relatively more severe clinical course. The above mentioned led us to the conclusion that prior to the universal newborn screening there was underestimation of the “true” incidence of IMDs. Also, we assume that even now there is still an underestimation of the “true” incidence, given the fact that many of these diseases are still not diagnosed (for example, in cases of early mortality or in cases of subtle disease that has not included in the newborn screening yet) and most of them are not included in the extended newborn screening program.

The high prevalence of certain diseases in different Bedouin clans as part of the total Bedouin population led to an additional genetic consultation to the different clans accordingly. Raz et al. has revealed that although there is growing awareness of the association between cousin or other consanguineous marriage and genetic diseases including IMD, due to traditional aspects that involve solidarity, preservation of family property, social protection for women and parental authority there is even nowadays a high rate of intrafamilial marriages [34]. Another important factor that counterbalances the importance of underreporting that is common in the Bedouin community and should be taken into consideration is the difference in number of births between the Bedouin women and Jewish women in the Negev area, with a total fertility rate of 7.28 per Bedouin woman versus total fertility rate of 2.75 per Jewish woman [35]. Therefore, premarital carrier matching, which is already performed in ultra-orthodox communities can be a major factor in reducing the prevalence of IMD in this community.

## Conclusion

IMDs are significantly more common in the consanguineous Bedouin compared with the relatively non-consanguineous Jewish population of Southern Israel. In the Bedouin population, IMD should not be considered as "rare" diseases since at least 1/1000 live birth has an IMD. Health policy should take into account these data.

## Methods

### Settings

The Negev district of Southern Israel was inhabited by 643,000 individuals in 2012 according to the Israeli Central Bureau of Statistics [36]; of these, 64% were Jews. Of the whole Negev population, 12% were children < 4 years old (77,000 children); among them 48% were Jewish children. Between 1990 and 2017 there were 187,049 Bedouin live birth and 206,723 Jewish live birth; overall of 393,772 live birth. Those numbers served as denominator for incidence calculations.

Since May 2008 Israel established universal Newborn Screening program which includes in the IMD part: Phenylketoniria (PKU), MSUD, Tyrosinemia type I, Homocystinuria, Methylmalonic acidemia, Propionic academia, Glutaric aciduria type I, Medium Chain Acyl-Carnitine dehydrogenase Deficiency (MCADD), Very Long Chain Acyl-CoA dehydrogenase Deficiency (VLCAD) [29].

Bedouin and Jewish children in the southern of Israel have access to the same medical services. Medical insurance in Israel is free and universal therefore there are no financial barriers in the availability of hospital services. The Soroka University Medical Center (SUMC) is the only hospital that serves the entire region. All births and evaluation of suspected IMDs are referred to SUMC. This exclusivity enables us to calculate incidence of IMDs according to ethnicity.

### Study design

This is a retrospective study conducted at SUMC from 1990 to 2017. The following inherited metabolic diseases were evaluated:

- Aminoacidopathies—Maple Syrup Urine Disease (MSUD), Non-Ketotic Hyperglycinemia;
- Peroxisomal diseases—X-linked Adrenoleukodystrophy (ALD), Zellweger disease;
- Sphingolipidosis—Niemann–Pick disease type 1;
- Organic acidemia—Glutaric Aciduria; Propionic acidemia; Isovaleric acidemia
- Fatty acid oxidation disease—Multiple Acyl-Coa Dehydrogenase deficiency (MADD), Medium chain Acyl-Coa Dehydrogenase deficiency (MCAD), Very long-chain acyl-CoA dehydrogenase deficiency (VLCAD), Carnitine Palmitoyl-transferase 1A (CPT 1A), Carnitine Palmitoyl-transferase 2 (CPT 2), Long chain 3-hydroxyl-CoA Dehydrogenase deficiency (LCHAD);
- Lysosomal storage diseases—Mucopolysaccharidosis types I, III,IVa, VI
- Glycogen Storage diseases—type 0, 1A, 1B, 2, 3, 6, 9, 11.
- Mitochondrial diseases—Complex 1 deficiency, Complex 3 deficiency, Complex 5 deficiency, Pyruvate dehydrogenase deficiency types 1A and Dihydrolipoamide dehydrogenase deficiency, Kearns Sayre syndrome, Mitochondrial Neuro-Gastrointestinal encephalopathy disease (MNGIE), Trans-Membrane protein 70 deficiency (TMEM 70), Mitochondrial DNA depletion.

Details were extracted from the computerized data of metabolic unit in SUMC.

### Statistical analysis

Incidence rates were calculated per specific disease in each ethnic origin separately and compared using Chi square test of Fisher exact test as appropriate. Comparison between the ethnic origin and incidences were performed. 95% confidence interval was calculated; *P* value of < 0.05 was considered significant. This study was approved by SUMC ethical committee.

## Data Availability

The datasets used and/or analyzed during the current study are available from the corresponding author for reasonable request.

## Abbreviations

IMD: Inherited metabolic disorders
MCADD: Medium chain acyl-carnitine dehydrogenase deficiency
VLCAD: Very long chain acyl-carnitine dehydrogenase deficiency
